# Cross-domain neural collaborative filtering for personalized herbal prescription recommendation

**DOI:** 10.1186/s13020-025-01294-9

**Published:** 2026-01-28

**Authors:** Xin Dong, Wansong Zhang, Kuo Yang, Lei Zhang, Runshun Zhang, Juxian Tang, Xinyu Wang, Rouye Huang, Dejiang Ji, Gaxi Ye, Xuezhong Zhou

**Affiliations:** 1 Department of Artificial Intelligence, Beijing Key Laboratory of Traffic Data Mining and Embodied Intelligence, School of Computer Science and Technology, Beijing Jiaotong University, Beijing, 100044 China; 2 National Data Center of Traditional Chinese Medicine, China Academy of Chinese Medical Sciences, Beijing, 100700 China; 3 Guang’anmen Hospital, China Academy of Chinese Medical Sciences, Beijing, 100053 China; 4 Department of Acupuncture and Moxibustion, Ningxia Hui Autonomous Region Hospital of TCM, Yinchuan, 750021 China

**Keywords:** Herbal prescription recommendation, Cross-domain learning, Neural collaborative filtering

## Abstract

**Objective:**

Herbal prescriptions hold significant importance in Traditional Chinese Medicine (TCM) diagnosis and treatment, embodying millennia of clinical case summaries and wisdom. Despite numerous proposed methods for herbal prescription recommendation (HPR), significant challenges persist due to the lack of comprehensive clinical data, particularly regarding the relationships between symptoms and herbs. This scarcity poses considerable hurdles for effective HPR modeling.

**Methods:**

In this study, we introduced a novel herbal prescription recommendation framework with cross-domain neural collaborative filtering (termed PresRecCDL). The cross-domain learning mechanism is introduced to learn the noise-reduced cross-domain features of herbs and symptoms in the unified space, which alleviated the sparsity of data, and the neural collaborative filtering is utilized to carry out prescription recommendations.

**Results:**

Comprehensive experiments demonstrate the superiority of the proposed PresRecCDL model over the SOTA model. The effectiveness of each module in PresRecCDL and model robustness are validated by the ablation and hyper-parameter tuning experiments, respectively. The case study based on network pharmacology further validates the effectiveness of the proposed approach, particularly its scientific rigor and feasibility at the molecular mechanism level.

**Conclusion:**

This study contributes to enhancing the performance of the HPR model, ultimately benefiting the efficiency and precision of clinical treatment.

## Introduction

In the past millennium, traditional Chinese medicine (TCM) has played a crucial role in safeguarding the health of the Chinese populace [1]. Its influence has also extended globally, offering valuable assistance in various health contexts. However, recent years have witnessed a surge in demand for high-quality TCM treatments amid challenges posed by limited medical resources [2]. Meeting these escalating needs calls for an urgent enhancement in diagnostic and treatment efficacy, alongside a refinement of professional skills among practitioners.

Central to the effectiveness of TCM are the quality and precision of herbal prescriptions, which directly reflect the proficiency of TCM practitioners. The accumulation of extensive clinical data in recent years offers a promising opportunity to leverage the experiential wisdom of doctors. This can significantly bolster the efficiency of TCM diagnosis and treatment, thereby providing invaluable support to clinicians in their therapeutic endeavors [3]. Currently, researchers have proposed herbal prescription recommendation (HPR) methods in this area [4–7], and these methods achieve relatively acceptable results, but they still suffer from some critical problems, including the following aspects.

**Fig. 1:**
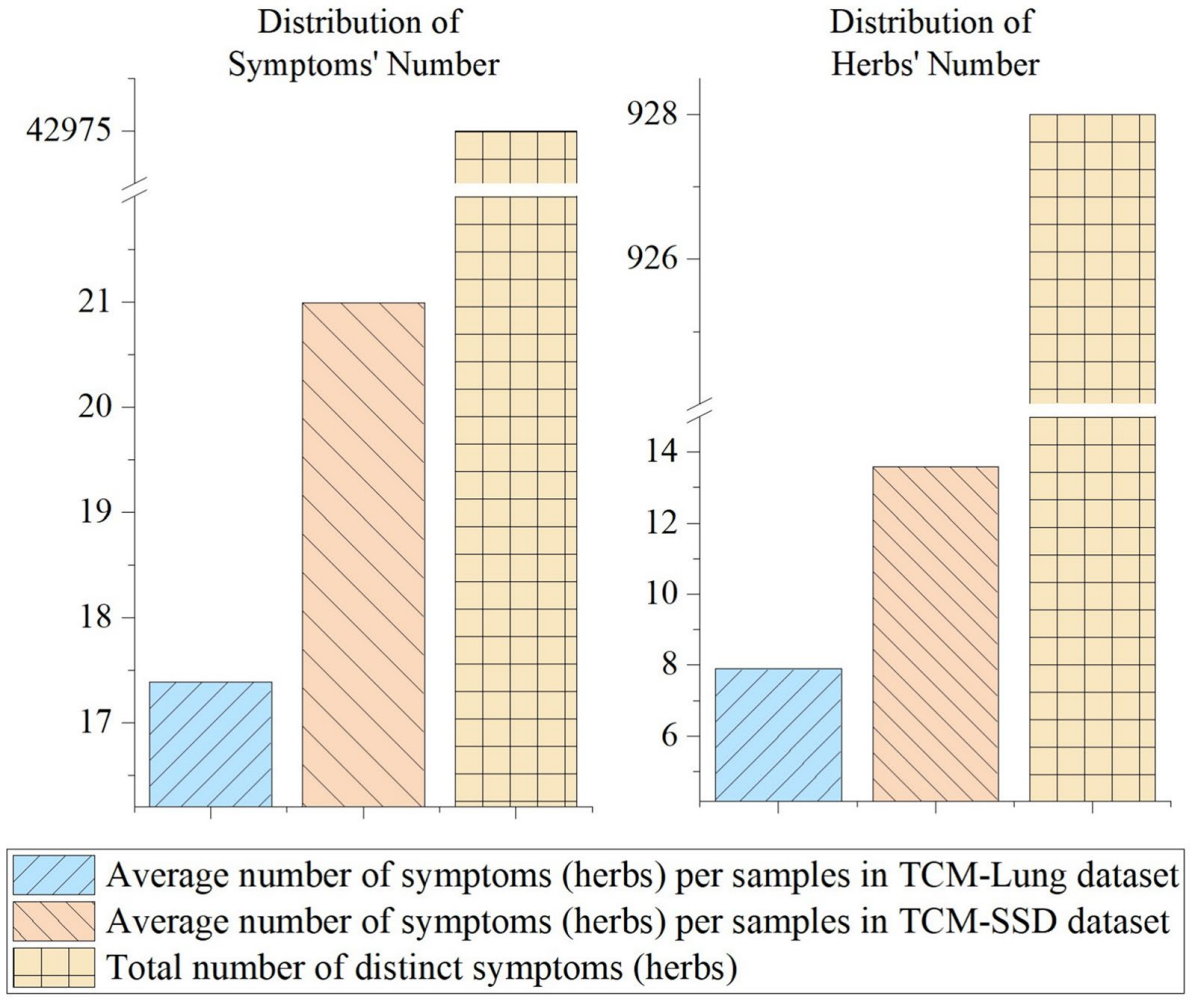
Sparsity of clinical dataset. This Figure records the distribution of symptoms (left) and herbs (right) for two datasets (*TCM-Lung* and *TCM-SSD*).

- **Sub-optimal performance.** The performance of existing models cannot be effectively applied in real-world clinical scenarios. For instance, in works like [4, 6], the Top@5 metric on their dataset has not exceeded 0.3. In other words, out of the top five predicted herbs, on average, only about 1–2 are correct. A model with such accuracy is not acceptable to medical professionals for real-world scenarios [7].
- **Sparsity of existing dataset.** In both TCM clinical data and HPR modeling, symptom information typically serves as the features of patient samples, while the prescribed herbal prescriptions constitute the labels. Following the definition of data sparsity in recommender systems and label density in multi-label learning [8, 9], we quantify the sparsity of the symptom–herb dataset from two perspectives. Through analyzing the quantity of symptoms and herbal prescriptions, we observed significant *sample-level sparsity*^1^ within TCM clinical datasets (Fig. 1). For the stomach and spleen disease dataset (*TCM-SSD* [37]), herb sparsity $S_{h}$ is 97.74%, symptom sparsity $S_{s}$ is 99.97%; for the lung disease dataset (*TCM-Lung* [7]), herb sparsity $S_{h}$ is $98.13\%$, symptom sparsity $S_{s}$ is $99.98\%$. Such sparsity may hinder feature learning and representation, thereby degrading the recommendation performance of HPR models.

Therefore, how to deal with the sparsity of clinical data and make an herbal prescription recommendation based on the characteristics of clinical datasets of different diseases is an urgent problem to be solved. It is gratifying to observe that the advancement of recommender system algorithms has led us to recognize the potential application of recommendation algorithms in the development of HPR models [10]. The popular cross-domain recommendation (CDR) technique in recent years is to get better features by learning cross-features between different domains to relieve the data sparsity, and to prevent an increasingly narrow range of items from being recommended to users [11]. Inspired by the CDR, we apply cross-domain learning technology to the HPR modelling for relieving data sparsity and improving the performance.

**Fig. 2:**
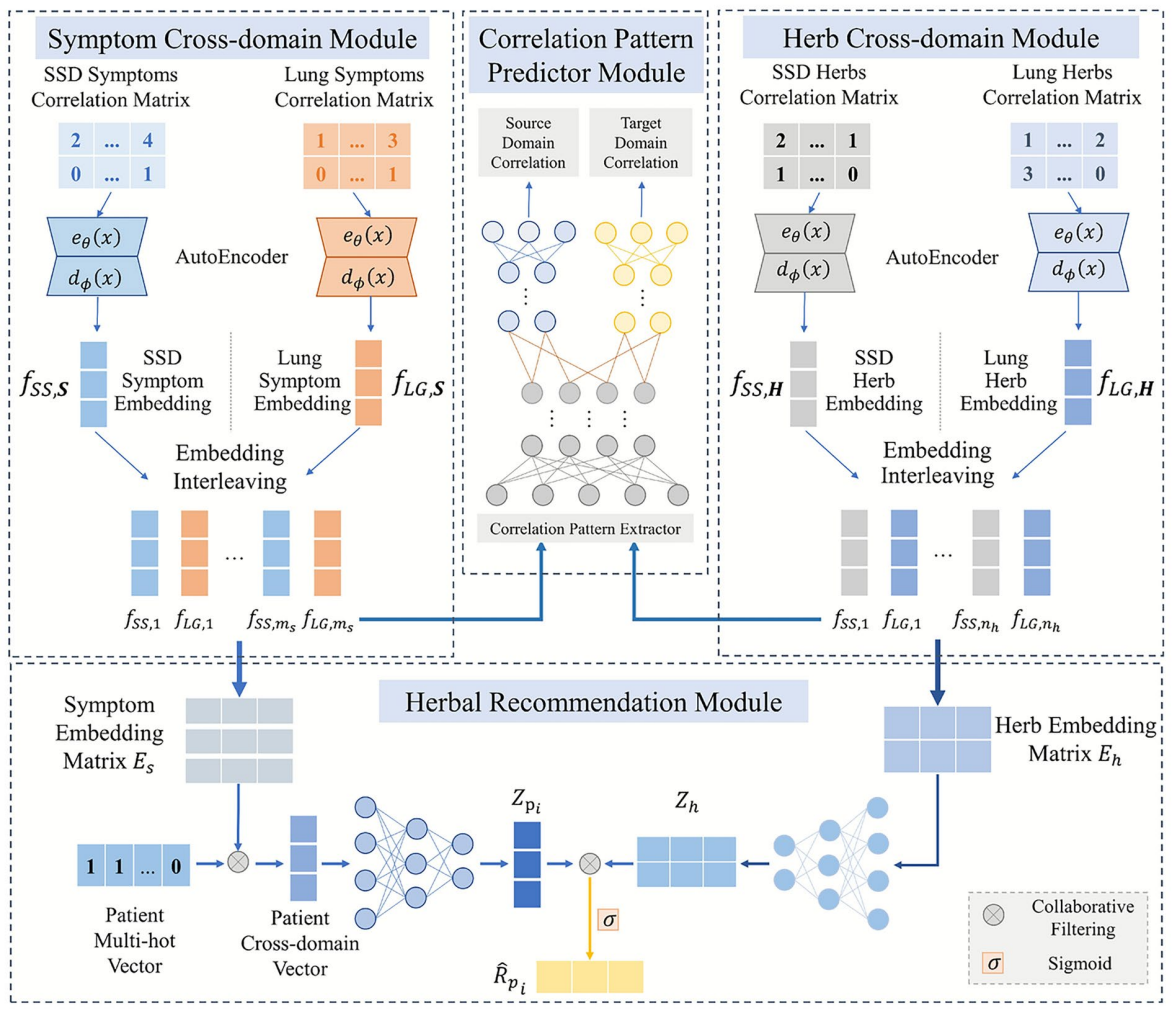
Architecture diagram of PresRecCDL. The proposed PresRecCDL consists of four main modules for cross-domain symptom-herb representation learning and herbal recommendation.

In this study, we propose an herbal prescription recommendation framework with cross-domain neural collaborative filtering (termed PresRecCDL Fig.2). Cross domain adversarial learning mechanism is introduced to learn noise-reduced cross-domain features of herbs and symptoms in a unified space. Neural collaborative filtering network is designed to conduct herbal prescription recommendations, aiming to enhance the accuracy and effectiveness of recommendations by effectively integrating cross-domain features. We conducted experiments on the *TCM-Lung* and the *TCM-SSD* dataset. Comprehensive experiments demonstrate the superiority of the proposed PresRecCDL model over the SOTA model. The effectiveness of each module in PresRecCDL and model robustness are validated by the ablation and hyper-parameter tuning experiments. The case study based on network pharmacology provides strong evidence for the effectiveness of the proposed approach, particularly its rationality and reliability at the molecular mechanism level.

Our contribution includes the following aspects.

- In order to alleviate the problem of data sparsity in HPR modeling, we introduced cross-domain learning mechanism to HPR modelling, and proposed PresRecCDL with cross-domain neural collaborative filtering. To the best of our knowledge, this work is the first to integrate CDR to alleviate the problem of data sparsity in TCM herbal prescription recommendation.
- In the proposed PresRecCDL, the cross-domain learning mechanism is utilized to learn the cross-domain features of herbs and symptoms after noise reduction in a unified space, the neural collaborative filtering network is designed for achieving accurate prescription recommendation. The cross-domain neural collaborative filtering enables the proposed PresRecCDL to have better representation and prediction capabilities, thereby improving prediction performance of herbal prescriptions.
- We empirically validated PresRecCDL against existing models on the two datasets, showcasing its superior performance in predicting herbal prescriptions. Additionally, we conducted a thorough analysis of ablation study and hyper-parameters such as the impact of CDL based symptom and herb embedding, dropout ratio, weight of regularization and embedding dimensions. Case studies based on network pharmacology offered valuable insights into the adaptability and robustness of the proposed innovative approach.

## Related work

### Herbal prescription recommendation models

In recent years, scholars have proposed numerous HPR methods, which can be divided into two categories: topic model-based HPR and deep learning-based HPR.

In the medical domain, one can regard a medical record as a "document", view treatment activities and patient features as "words", and treatment patterns as "topics" [12]. Therefore, topic model techniques can be suitably utilized in modeling HPR. Blei [13] made a direct analogy between a kind of data and documents, while Fei et al. [14] assumed each image is a group of "visual words" and shows a combination of visual patterns (topics), Chen et al. [15] incorporated linguistic knowledge about words into topic modeling in several works, Huang et al.[12] used topic modeling to discover potential treatment patterns of clinical pathways [12], Li et al. [16] constructed herb network using a method called Distance-based Mutual Information Model to identify useful relationships among herbs in numerous prescriptions, He et al. [17] proposed an approach that could discover herbal functional groups from a large set of prescriptions recorded in TCM books. However, due to the complexity of the association between herbs and symptoms, the application of topic model techniques in HPR modeling often involves high computational complexity. As a result, related work on topic models has gradually decreased in recent years.

With the development of deep learning techniques, many deep learning-based HPR models have been proposed to address problems that previous methods couldn’t solve. A meta-path-guided graph attention network [18] was proposed, which combines the pharmacology of modern medicine with TCM knowledge, and transforms TCM from empirical medicine to evidence-based medicine. An integrated clustering and ranking information network method was also proposed to optimize herbal prescription recommendations [19], Ji et al. [20] introduced a TCM knowledge graph to enrich the input corpus, constructing a knowledge graph-enhanced multi-graph neural network architecture. To address the poor features of constructed herbs, Liu et al. [21] proposed the method of interactive knowledge graph to enhance the association between herbs and symptoms. Zhou et al. [22] proposed an intelligent prescription recommendation system based on deep learning that fused phenotypic and molecular information, extracting features of herbal prescriptions through network embedding fused with molecular information, Qin et al. [23] proposed a recommendation algorithm based on mutual information clustering, Liu et al. [24] applied a pre-trained language model to the domain of chinese patent medicine, Zhao et al. [25] proposed a hybrid neural network architecture for TCM prescription generation—PreGenerator. However, the aforementioned work still faces issues such as data sparsity and suboptimal performance, making the formation of usable herbal prescription recommendations a key challenge.

### Cross domain recommendation

Cross-domain recommendation aims to enhance recommendation systems by integrating data from multiple domains, utilizing information from source domains to improve outcomes in the target domain and beyond [26]. Generally, cross-domain recommendation (CDR) can be divided into two primary models: collaborative filtering-based cross-domain recommendation (CFCDR) and content-based cross-domain recommendation (CBCDR).

CFCDR is suitable for scenarios where the source and target domains exhibit a certain degree of similarity, while CBCDR is more appropriate when there is a significant correlation between the domains. Early implementations of CFCDR relied on neighborhood-based solutions [27], but these methods were soon supplanted by Matrix Factorization (MF). However, MF-based CFCDR models often depend on the density of data in either the source or target domain [28], and they struggle with processing nonlinear data. To mitigate MF’s dependency on data density, Chun et al.[29] proposed a cross-domain recommendation algorithm for quadratic collaborative filtering, which leverages a similarity matrix to address the sparsity in both the source and target domains. Additionally, M. Enrich proposed utilizing a separate domain for item recommendations to users [30].

To overcome the limitations of CFCDR, CBCDR models were introduced. CBCDR primarily relies on the content and behavioral attributes of users and items, and does not require other user information, enabling recommendations even in cold start scenarios (i.e., new users or items). Several methods have been developed to handle cold start in CBCDR. For instance, Liu et al. proposed using TrAdaBoost to select potentially recommendable items for users, followed by a nonparametric pairwise clustering algorithm to refine the recommendations [31]. Additionally, to address the cold start problem when there is an overlap in attributes between the user and item domains, Zhu et al. [32] suggested increasing the information considered from the source domain to enhance recommendation accuracy.

In the context of herbal prescription recommendation, challenges such as insufficient datasets and data sparsity are prevalent. To address these issues, we propose employing cross-domain learning, specifically CBCDR, for herbal prescription recommendation. This approach aims to mitigate data sparsity and achieve improved recommendation outcomes.

## Material and methods

### Framework of PresRecCDL

The framework of PresRecCDL, illustrated in Fig. 2, comprises four main modules: (1) Symptom Cross-domain Module (SCM): This module generates cross-domain symptom representations from clinical data’s symptom co-occurrence matrix using an autoencoder, combining *TCM-SSD* and *TCM-Lung* for downstream patient representation. (2) Herb Cross-domain Module (HCM): Similar to SCM, this module processes a herb co-occurrence matrix using an autoencoder to generate herb representations for subsequent processing. (3) Correlation Pattern Predictor Module (CPM): Herb and symptom matrices are input into a fully connected neural network-based correlation pattern predictor to extract effective cross-domain embeddings. (4) Herbal Recommendation Module (HRM): By integrating the symptom and herb features generated by the aforementioned modules, the HRM module is designed to perform the herb recommendation task based on the comprehensive representations of patients and herbs, utilizing the neural collaborative filtering technology and resulting in predicted probabilities for each herb for the patient.

#### Symptom cross-domain module and herb cross-domain module

To form symptom and herb features based on cross-domain learning and alleviate the issue of data sparsity, the primary task of the proposed PresRecCDL is to learn cross-domain features of symptoms and herbs based on clinical data co-occurrence relationships. Therefore, we implemented SCM and HCM separately to learn herb and symptom features from *TCM-SSD* and *TCM-Lung*. First, we extracted $m_{s}$ distinct symptoms and $n_{h}$ distinct herbs from the two datasets, i.e., *TCM-SSD* and *TCM-Lung*. We generated the dictionaries of symptoms and herbs as $\left\langle \left[ s_{1},\ldots ,s_{m_{1}} \right] ,\left[ h_{1},\ldots ,h_{n_{1}} \right] \right\rangle$ for *TCM-Lung*, $\left\langle \left[ s_{1},\ldots ,s_{m_{2}} \right] ,\left[ h_{1},\ldots ,h_{n_{2}} \right] \right\rangle$ for *TCM-SSD*, where *s* and *h* denote symptoms and herbs, respectively. We then conducted a co-occurrence calculation to establish the relationships between symptoms and herbs, obtaining correlation matrices for symptoms ($M_{H\Rightarrow S}$) and herbs ($M_{S\Rightarrow H}$), as follows,

1 $$\begin{aligned} & M_{H\Rightarrow S}=\left[ C_{ij} \right] _{n_{h}\times max\left\{ m_{1}, m_{2} \right\} } \end{aligned}$$

2 $$\begin{aligned} & M_{S\Rightarrow H}=\left[ C_{ij} \right] _{m_{s} \times max\left\{ n_{1}, n_{2} \right\} } \end{aligned}$$

where $C_{ij}$ is calculated by the following formulas,

3 $$\begin{aligned} & C_{ij}=\sum t_{h_{i},s_{j}} \end{aligned}$$

4 $$\begin{aligned} & t_{h_{i},s_{j}}=\left\{ \begin{aligned} 1&\quad \text {if }(h_{i},s_{j})\text { co-occurs in one patient};\\ 0&\quad \text {otherwise}\\ \end{aligned} \right. \end{aligned}$$

Through the above computation process, calculations are performed on *TCM-SSD* and *TCM-Lung* separately, resulting in co-occurrence matrices of symptoms and herbs for the two datasets.

The subsequent process involves cross-domain feature learning based on the co-occurrence matrices from the two datasets. It is important to note that since the two datasets involve two different diseases, the symptom expressions vary, but the herbs have commonalities [33]. Therefore, the cross-domain common component is herbs. We used *TCM-SSD* as the source domain and *TCM-Lung* as the target domain, conducting the cross-domain learning task based on an AutoEncoder structure [34]. We used two separate AutoEncoders for cross-domain feature learning: the H-AutoEncoder for learning herb features and the S-AutoEncoder for learning symptom features.

H-AutoEncoder and S-AutoEncoder take the partially observed correlation vectors for each herb (symptom), $y_{h}$, as input. As an example in forming herb embedding, $y_{h}$ represents the herb embedding in $M_{S\Rightarrow H}$, where $y_{h}=\left[ C_{ij} \right] _{n_{h}\times 1}$. Each vector is then mapped to a low-dimensional latent vector $\hat{y}_{h}$, as follows,

5 $$\begin{aligned} \hat{y}_{h} =f_{1}\left( W_{2}f_{2}\left( W_{1}y_{h}+b_{1} \right) +b_{2} \right) \end{aligned}$$

where $f_{1}\left( \cdot \right)$ and $f_{2}\left( \cdot \right)$ are activation function (ReLU is utilized in this study), $W_{1},W_{2}$ are corresponding weights matrix of the AutoEncoder, $b_{1},b_{2}$ are corresponding biases of the AutoEncoder. We used the regularized square loss to optimize the AutoEncoder as follows,

6 $$\begin{aligned} Loss_{CDL} = {\textstyle \sum _{s=1}^{S}}\left\| \hat{y_{h}}-y_{h} \right\| _{O}^{2} + \alpha \left( \left\| W_{1} \right\| _{F}^{2} +\left\| W_{2} \right\| _{F}^{2} +\left\| b_{1} \right\| _{2}^{2} +\left\| b_{2} \right\| _{2}^{2} \right) \end{aligned}$$

where $\left\| \cdot \right\| _{F}^{}$ denotes matrix Frobenius norm, $\left\| \cdot \right\| _{2}^{}$ denote vector L2-norm respectively, *O* denotes the observed ratings in each vector $y_{h}$, $\alpha$ controls the regularization strength. Finally, symptom embedding $\left( E_{s} \right)$ and herb embedding $\left( E_{h} \right)$ are generated by interleaving herb vectors and symptom vectors obtained through cross-domain learning.

#### Correlation pattern predictor module

After training the SCM and HCM modules, we used the obtained parameters $W_{1}$ and $b_{1}$ to compute $f_{h} = f_{2}(W_{1} y_{h} + b_{1})$, representing the low-dimensional latent vectors for herbs and symptoms in both domains. Next, we leveraged these vectors for cross-domain feature interaction to extract correlation rating patterns from the latent space, thus designing the CPM module.

The CPM module takes the vector $f_{h}$ as input and implements a correlation pattern predictor network based on a deep feed-forward neural network. This network consists of a rating pattern extractor, a domain classifier, and a predictor for rating estimation tasks. The rating predictor is designed using the parameter set $\theta _{r}$ to reconstruct the rating vectors for *TCM-SSD* and *TCM-Lung* separately. Additionally, the rating pattern extractor is developed using a fully connected neural network with the parameter set $\theta _{f}$ for feature extraction. The domain classifier is created using another neural network with the parameter set $\theta _{c}$ to predict the corresponding domain labels. During training, we optimized the rating predictor, rating pattern extractor, and domain classifier to identify domain-learning features. L2 regularization is denoted by $\lambda$ as follows,

7 $$\begin{aligned} L\left( \theta _{f },\theta _{r },\theta _{c } \right) =loss_{pred}\left( \theta _{f }, \theta _{r }\right) -\mu loss_{pred}\left( \theta _{c } \right) + \lambda R \end{aligned}$$

where $\mu$ is hyperparameter, $R= \left\| \theta _{f } \right\| ^{2} +\left\| \theta _{r } \right\| ^{2} +\left\| \theta _{c } \right\| ^{2}$ is the regularizer term to alleviate overfitting. Through the implementation of the above process, we obtained cross-domain features for herbs and symptoms. The formation of these features helps alleviate the problem of data sparsity, thereby improving the development of patient characteristics and the herb recommendation task.

#### Herbal recommendation module

Through the previous cross-domain learning modules, we obtained cross-domain features of symptoms and herbs based on *TCM-Lung* and *TCM-SSD*. The next step is herb recommendation, which we achieved with the HRM module based on neural collaborative filtering. First, we utilized one-hot coding with symptom embedding for the prescription’s symptom set [35]. Then, a comprehensive embedding for the symptom set was obtained, as follows,

8 $$\begin{aligned} Z_{p_{i}} =ReLU\left( MLP\left( avg\_ pool \left( P_{i}\cdot E_{s} \right) \right) \right) \end{aligned}$$

where $P_{i}$ is a multi-hot vector representing the patient’s symptoms, $ReLU$ is the activation function, $MLP$ is the multi-layer perception, and $avg\_pool$ is the pooling layer. Simultaneously, previous herb embedding $E_{h}$ are computed to obtain comprehensive herb embedding, as follows,

9 $$\begin{aligned} Z_{h} =ReLU\left( MLP\left( E_{h} \right) \right) \end{aligned}$$

and finally, the neural collaborative filtering process based on the comprehensive representation of patients and herbs is implemented as follows,

10 $$\begin{aligned} \hat{R}_{p_{i} } =sigmod\left( Z_{p_{i}} \cdot Z_{h} \right) \end{aligned}$$

and the obtained $\hat{R}_{p_{i}}$ represents the predicted probability of each herb for each patient. Finally, we used BCELoss [36] to optimize the PresRecCDL, as follows,

11 $$\begin{aligned} Loss_{HR}=- \left[ y_{i}\cdot \log _{}{y_{i}^{\prime } + \left( 1-y_{i} \right) \cdot \log _{}{\left( 1-y_{i}^{\prime } \right) } } \right] \end{aligned}$$

where $y_{i}^{}$ is the actual label of herb, $y_{i}^{\prime }$ is the predicted herb. Through the above process, guided by cross-domain representations of symptoms and herbs, we ultimately obtained the predicted probabilities of patients for candidate herbs, thus achieving the herbal recommendation process.

### Experimental settings

#### Dataset

In this experiment, we used the *TCM-Lung* [7] and *TCM-SSD* (utilized in [37]) from two clinical datasets, with 17,593 samples in the *TCM-SSD* and 14,948 samples in the *TCM-Lung*. The herbal prescription involved the utilization of 14,948 pieces of *TCM-Lung* for learning, divided into training and test sets in an 8:2 ratio. The training set comprised 11,958 pieces, while the test set contained 2,990. Similarly, for *TCM-SSD*, 17,593 pieces were employed for learning, with the same division way for training and test sets. The training set encompassed 14,074 pieces, while the test set comprised 3,519.

**Fig. 3:**
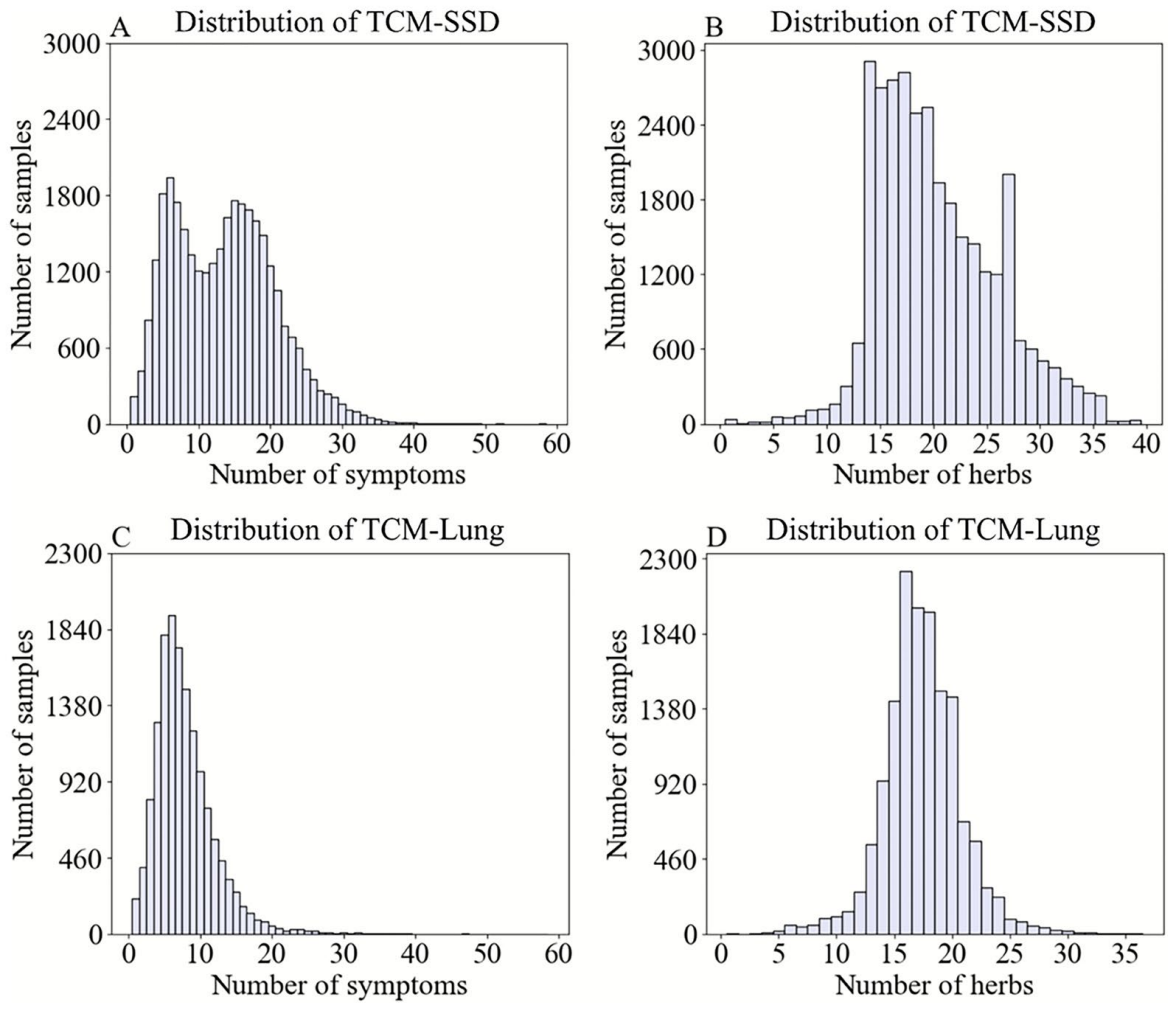
Performance of herb distribution and symptom distribution across datasets. (**A**-**B**) Symptom and herb count distributions in the *TCM-SSD* dataset. (**C**-**D**) Symptom and herb count distributions in the *TCM-Lung* dataset.

To verify the sparsity of the data, we conducted a quantitative analysis of *TCM-Lung* and *TCM-SSD* (Fig. 3). It reveals that most symptoms are concentrated between 1 and 30 for *TCM-SSD* and between 1 and 20 for *TCM-Lung*. The most frequent symptom occurs 3,251 times for *TCM-SSD* and 1,939 times for *TCM-Lung*. Similarly, the majority of herbs are concentrated between 5 and 36 for *TCM-SSD* and between 5 and 25 for *TCM-Lung*. The most frequent herb appears 2,819 times for *TCM-SSD* and 2,460 times for *TCM-Lung*. On average, each sample contains 13.58 symptoms for *TCM-SSD* and 17.39 symptoms for *TCM-Lung*, while the average number of herbs per sample is 20.98 for *TCM-SSD* and 7.89 for *TCM-Lung*. There are 285 overlapped herbs between *TCM-Lung* and *TCM-SSD*, along with 328 overlapped symptoms. The herb set consists of 928 distinct herbs, and the symptom set contains 42,975 distinct symptoms.

#### Baselines

In our experiment, we used several models as the baselines, as follows:

- LinkLDA [38] is a model that relies on the assumptions of the four levels of population, subject, latent variables, and sampling method to process mixed data.
- BlockLDA [39] is to share information between the block model and the text model, Block-LDA is to build a link matrix, then build a generative model to make inferences, and finally generate herbs.
- Link-PLSA-LDA [40] combines PLSA and LDA in the same framework to address the effects of a completely unsupervised approach to specific symptoms.
- ATM [41] is to build an herb-symptom model and then use the latent Dirichlet allocation [42] to get a corpus of an herb set, along with the symptom Relevance to Herb.
- PTM [43] is an herbal recommendation for treatment methods obtained through the mapping of symptoms in the field of herb, but because of the duplication of treatment methods, the relationship between symptom and herb cannot be directly generated.
- TCMPR [44] is an herbal recommendation method based on the symptom item mapping method of sub-network (SSTM), which is an herbal recommendation method that combines the original symptom words of patients with SSTM.
- KDHR [45] first constructs the herb knowledge graph, and uses GCN and multilayer perceptron (MLP) to integrate the information to obtain a node feature representation with richer information and less noise, and finally outputs the recommended method of predicted herbs.

Our model is based on the TensorFlow2 framework and runs on a computer with the NVIDIA GeForce RTX 3060 Laptop GPU for GPU-accelerated training, CPU is 11th Gen Intel(R) Core(TM) i7-11800H @ 2.30GHz, memory is 16G.

#### Evaluation metrics for the HPR task

We adopted the Top@K evaluation metric for the prediction results in the test set. Assumed that the total number of samples contained in the test set is N. For the i-th test sample $d_{i}$, $R\left( i \right)$ represents the herb set predicted by the algorithm and $T\left( i \right)$ represents the real herb set of $d_{i}$. Then the precision rate (Precision@K), recall rate (Recall@K) and F1-score (F1-score@K) are as follows:

12 $$\begin{aligned} & Precision@K=\frac{ {\textstyle \sum _{i=1}^{N}\left| R\left( i \right) \cap T\left( i \right) \right| } }{\sum _{i=1}^{N}\left| R\left( i \right) \right| } \end{aligned}$$

13 $$\begin{aligned} & Recall@K=\frac{ {\textstyle \sum _{i=1}^{N}\left| R\left( i \right) \cap T\left( i \right) \right| } }{\sum _{i=1}^{N}\left| T\left( i \right) \right| } \end{aligned}$$

14 $$\begin{aligned} & F1-score@K=\frac{2*Precision@K*Recall@K}{Precision@K+Recall@K} \end{aligned}$$

#### Network pharmacology-based gene association analysis

In addition to the Top@K evaluation metric for the HPR task, we also performed association analysis between genes of predicted herbs and symptoms using network pharmacology techniques, focusing primarily on the following three metrics. The $S_{AB}$, Z-score, and Average Shortest Path Length (ASPL) metrics are used to evaluate the degree of association between two molecular networks. Let $A$ represent the molecular set of disease $A$, and $B$ represent the molecular set of disease $B$. The formula for calculating $S_{AB}$ is as follows:

15 $$\begin{aligned} S_{AB} = d_{AB} - \frac{d_{AA} + d_{BB}}{2} \end{aligned}$$

where $d_{AA}$ and $d_{BB}$ represent the shortest distances between proteins within sets $A$ and $B$, respectively, and $d_{AB}$ denotes the shortest distance between protein pairs from $A$ and $B$. The formula for the Z-score is as follows:

16 $$\begin{aligned} NR_{zs}(A,B) = \frac{s - s^{\text {rand}}}{\sigma (s^{\text {rand}})} \end{aligned}$$

where $s$ represents the shortest distance between sets $A$ and $B$ on the PPI network, and $s^{\text {rand}}$ and $\sigma (s^{\text {rand}})$ represent the mean and standard deviation obtained from 10,000 random trials, respectively. $NR_{zs}(A,B)$ indicates the network relevance (NR) between sets $A$ and $B$. A negative Z-score, with smaller values, indicates stronger relevance. The formula for ASPL is as follows:

17 $$\begin{aligned} NR_{sp}(A,B) = \frac{\sum \limits _{i \in A} \sum \limits _{j \in B} \text {SPL}(i,j)}{|A| \cdot |B|} \end{aligned}$$

where SPL$(i,j)$ represents the shortest path length between protein $i$ in set $A$ and protein $j$ in set $B$.

We used the gene function enrichment interface in SymMap [46] to perform GO and pathway enrichment analysis, and calculated the significance scores and odds ratios (OR values) for the pathways. In addition, to observe the molecular network associations between the genes corresponding to the herbs predicted by our model and the genes related to the input symptoms, we conducted network visualization. Inspired by [47], we also performed 1,000 rounds of random computation to reflect the distribution of results under random conditions. Specifically, we randomly selected two sets of genes from the PPI network, with the same number of genes as the symptom-related and herb-related genes, respectively, and plotted the distribution of the number of interaction edges between these two sets over 1,000 repetitions. The significance P-value was then calculated using a binomial test [48].

## Results

In this section, we conduct comprehensive experiments to validate the performance of the proposed PresRecCDL model, including a comparison with the baseline model, an ablation study to assess the contribution of different modules, hyper-parameter tuning to evaluate sensitivity and robustness, and a case study to verify the model’s reliability.

### Overall comparison

In our experiment, PresRecCDL is compared with seven baseline models, as shown in Table 1, using Precision, Recall, and F1-score for top@5, top@10, and top@20. PresRecCDL consistently outperformed all other models. KDHR achieved suboptimal performance with 58.49% on P@5, 17.49% on R@5, and 26.80% on F1@5. In comparison, PresRecCDL reached 60.26% on P@5, 17.71% on R@5, and 27.38% on F1@5, showing notable improvements of 3.03% in P@5, 1.26% in R@5, and 2.16% in F1@5 over KDHR. As K increases, the number of considered herbs grows, leading to a decline in precision, an upward trend in recall, and an increasing trend in F1-score for all methods. The top four models in Table 1 are based on topic models, which suffer from insufficient high-level connections between herbs and symptoms. Unlike KDHR and TCMPR, which rely on external knowledge (symptom network), our proposed PresRecCDL is a more concise model that does not depend on any external knowledge.

**Table 1:** Performance comparison of herbal prescription recommendation models

Dataset	Model	P@5	P@10	P@20	R@5	R@10	R@20	F1@5	F1@10	F1@20
*TCM-Lung*	LinkLDA	0.5751	0.4992	0.4061	0.1672	0.2894	0.4698	0.2591	0.3664	0.4357
	BlockLDA	0.5718	0.4966	0.4050	0.1661	0.2880	0.4687	0.2474	0.3645	0.4345
	Link-PLSA-LDA	0.5684	0.4932	0.4064	0.1651	0.2858	0.4701	0.2559	0.3619	0.4359
	PTM	0.5826	0.5085	0.4083	0.1699	0.2948	0.4726	0.2631	0.3732	0.4381
	TCMPR	0.5889	0.5119	0.4114	0.1715	0.2966	0.4760	0.2657	0.3756	0.4413
	KDHR	0.5849	0.5201	0.4164	0.1749	0.3048	0.4872	0.2680	0.3843	0.4472
	PresRecCDL	**0**.**6026**	**0**.**5233**	**0**.**4173**	**0**.**1771**	**0**.**3068**	**0**.**4884**	**0**.**2738**	**0**.**3869**	**0**.**4501**
*TCM-SSD*	LinkLDA	0.6479	0.5069	0.4488	0.1302	0.2023	0.3555	0.2168	0.2892	0.3968
	BlockLDA	0.6602	0.5205	0.4612	0.1326	0.2077	0.3653	0.2208	0.2970	0.4077
	LinkPLSALDA	0.6585	0.5199	0.4641	0.1322	0.2074	0.3674	0.2202	0.2965	0.4101
	PTM	0.6596	0.4742	0.4362	0.1326	0.1887	0.3448	0.2208	0.2700	0.3851
	TCMPR	0.6934	0.6037	0.5024	**0**.**1518**	**0**.**2633**	**0**.**4399**	**0**.**2491**	**0**.**3667**	**0**.**4691**
	KDHR	0.6462	0.5818	0.4738	0.1440	0.2599	0.4188	0.2355	0.3593	0.4446
	PresRecCDL	**0**.**7355**	**0**.**6294**	**0**.**5152**	0.1486	0.2527	0.4098	0.2473	0.3606	0.4565

The best results are bolded, and the second-best results are underlined

### Ablation experiments

**Fig. 4:**
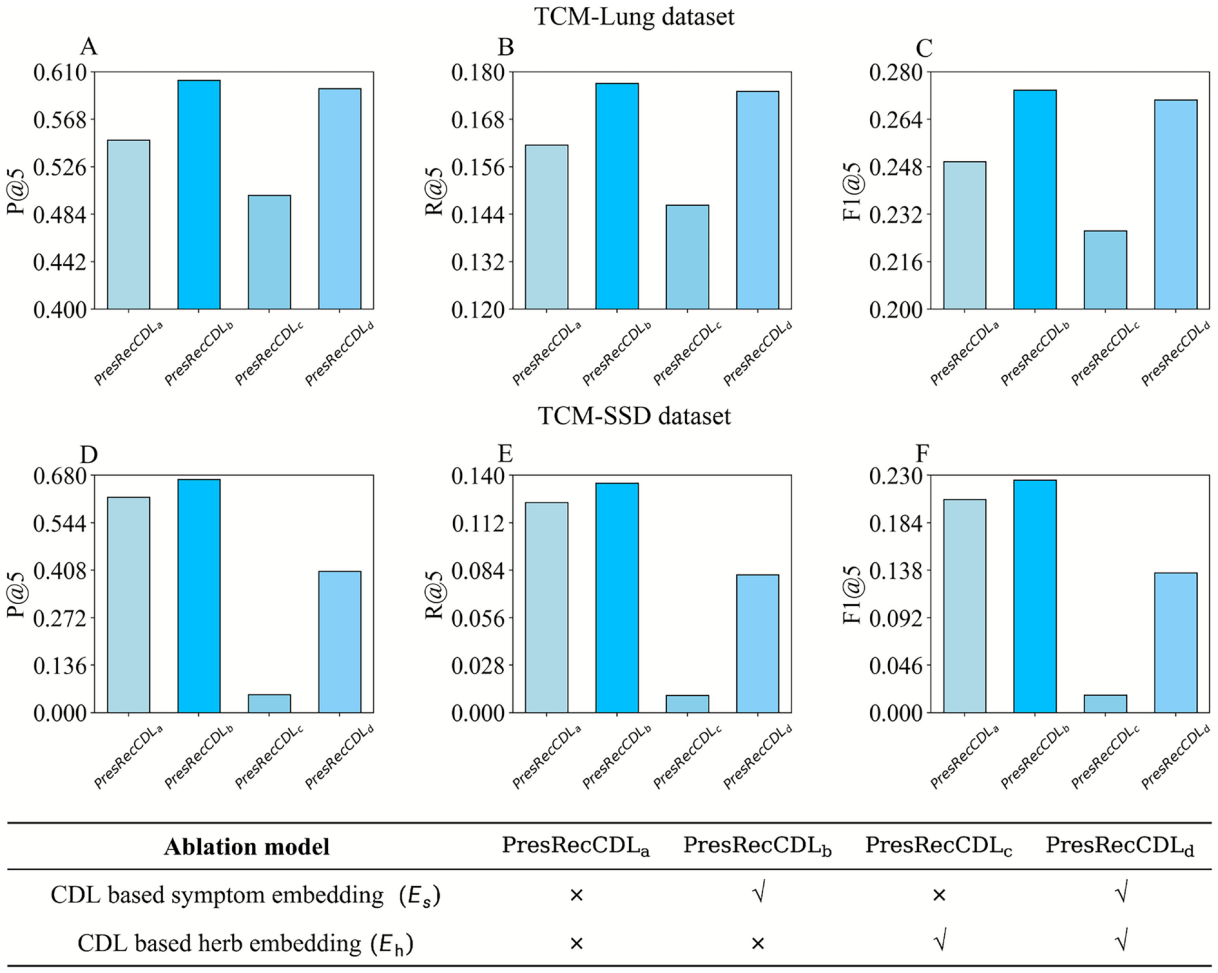
Performance of ablation experiment. In the figure, $PresRecCDL_a$/$PresRecCDL_b$/$PresRecCDL_c$/$PresRecCDL_d$ represent four experimental cross-domain combinations of features (the experimental design is presented in a table, where the adopted embedding are indicated by $\checkmark$). **A**–**C** are the experimental results on the *TCM-Lung* dataset, showing the results of P@5, R@5, and F1@5, respectively. **D**–**F** are the results on the *TCM-SSD* dataset.

To quantify the contribution of cross-domain learning in the PresRecCDL model, we conducted a series of ablation experiments encompassing four ablation models, varying in the inclusion of herb/symptom embeddings derived from cross-domain learning (as depicted in Fig. 4). Our observation are as follows:

First, model ${PresRecCDL}_b$, which incorporates symptom embeddings, exhibited the best performance. Compared to model $PresRecCDL_a$, which excluded both symptom and herb embeddings, model ${PresRecCDL}_b$ demonstrated a notable 3.01% improvement in overall performance, with a 1.49% increase in R@5 and a 2.31% boost in F1@5. This signifies a significant enhancement in P@5, R@5, and F1@5 metrics over the baseline when incorporating cross-domain learning.

Second, the results indicate that the combination of symptom embeddings and random herb embeddings yields the most favorable outcomes. Specifically, there was a significant 3.01% uplift in P@5 compared to the baseline, accompanied by a 1.49% increase in R@5 and a 2.31% surge in F1@5.

Third, the introduction of embeddings has enhanced the precision of PresRecCDL. Logically, this study provides evidence that cross-domain learning can enhance performance in sparse data recommendation scenarios. The dataset in works [4, 5] is sparser than the HPR dataset we utilized, whereas the *TCM-Lung* and *TCM-SSD* datasets are relatively concentrated. Given that the overlap ratio of herbs in these two datasets is significantly higher than the proportion of overlapping symptoms, the influence of herb embeddings on HPR is likely weaker than that of symptom embeddings.

Finally, the experimental results reveal that both herb and symptom embeddings can improve the efficacy of HPR. However, utilizing symptom embeddings alone for HPR outperforms the use of herb embeddings alone or the concurrent employment of both embeddings. This aligns with the characteristics of cross-domain learning for sparse data, suggesting that we can alleviate this as a single-domain recommendation problem [49].

### Results of hyper-parameter tuning

We fine-tuned key hyper-parameters in the PresRecCDL model, including Dropout, L2 regularization, embedding dimension, and MLP architecture. Hyper-parameters with minimal impact, such as the learning rate (1e−3) and Adam optimizer [50], were kept at default. Training was terminated early with an early stopping mechanism if the loss failed to improve for 20 epochs.

#### Performance comparison with different dropout

**Fig. 5:**
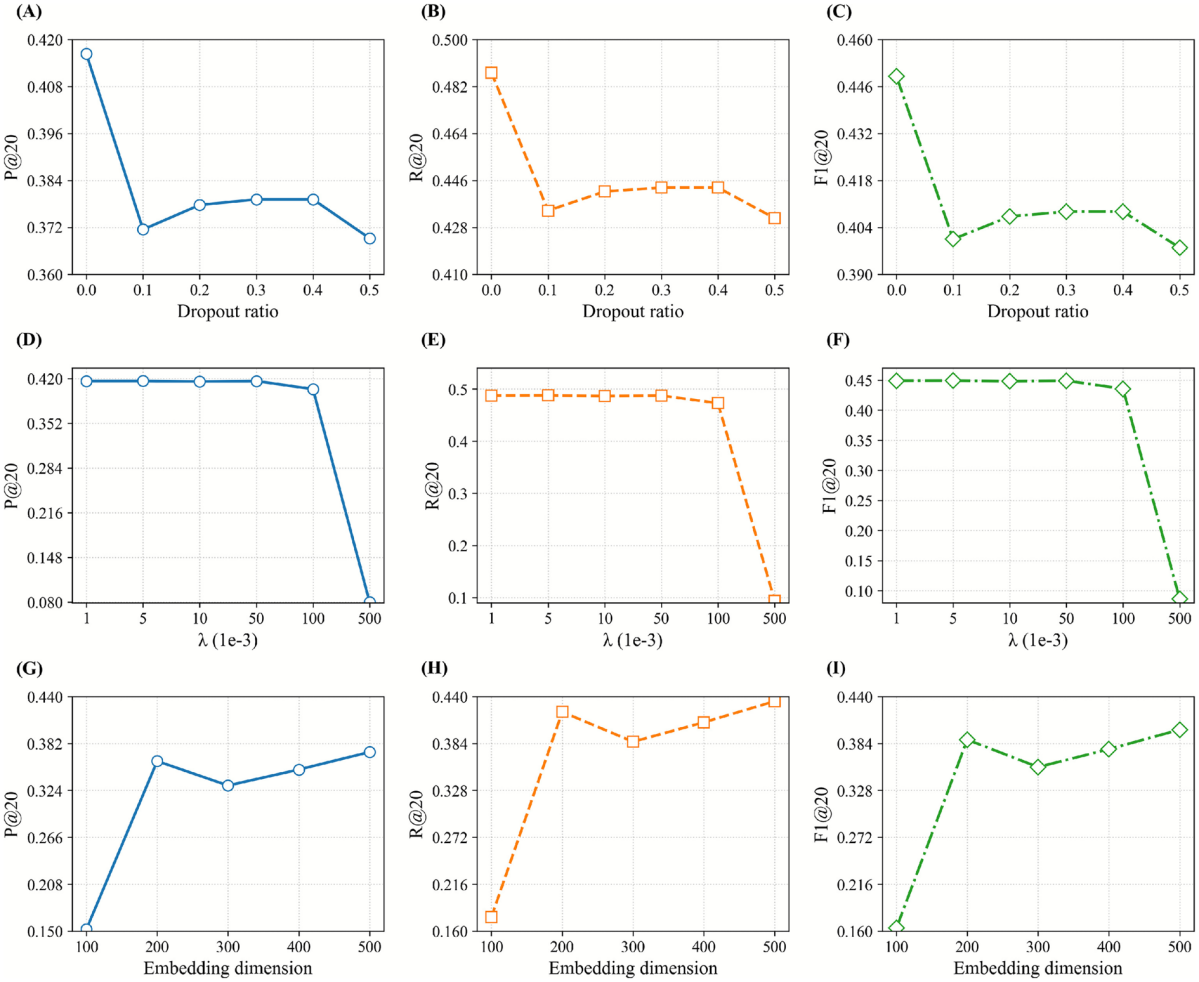
Performance of different dropout ratios, the weight of regularization ($\lambda$) of the model and dimensions of the embeddings obtained by cross-domain learning. (**A**-**C**) show how model performance changes with varying dropout ratios. (**D**-**F**) depict performance trends with respect to the regularization weight λ. (**G**-**I**) summarize the impact of embedding dimensions.

The performance of PresRecCDL decreased as a whole after adding Dropout, and the performance decreased first and then increased with the increase of Dropout (Fig. 5A–C). We compared the performance under different dropout ratios. The performance of the PresRecCDL decreases with the addition of Dropout. The Dropout had a decrease of 0.37–2.64% on P@20, 0.34–2.64% on R@20, 0.34–2.61% on F1@20. The results show that the performance of the PresRecCDL model shows a fluctuating decline after adding Dropout, thus leading us to infer that Dropout does not yield an optimal improvement for our model.

#### Performance comparison with different regularization hyper-parameter

We compared the effects of different regularization hyper-parameter $\lambda$ on HPR, the increase of L2 penalty term weight makes the performance of PresRecCDL decline in a fluctuating manner (Fig. 5D–F). P@20 decreased by 0.19–80.81%, R@20 decreased by 0.16–80.73%, F1@20 decreased by 0.17–80.78%. From the results, we found that PresRecCDL has better performance when the $\lambda$ is 0.001.

#### Performance comparison with different embedding dimension

We explore the feature dimension and set the dimensions to 100, 200, 300, 400, and 500. The results (Fig. 5G–I) demonstrates that as the increase of embedding dimension, the performance of PresRecCDL fluctuates when the dimension is increased from 100 to 500. Finally, we conclude that the embedding dimension of 500 is a relatively optimal choice.

#### Performance comparison with different layer number of MLP

**Fig. 6:**
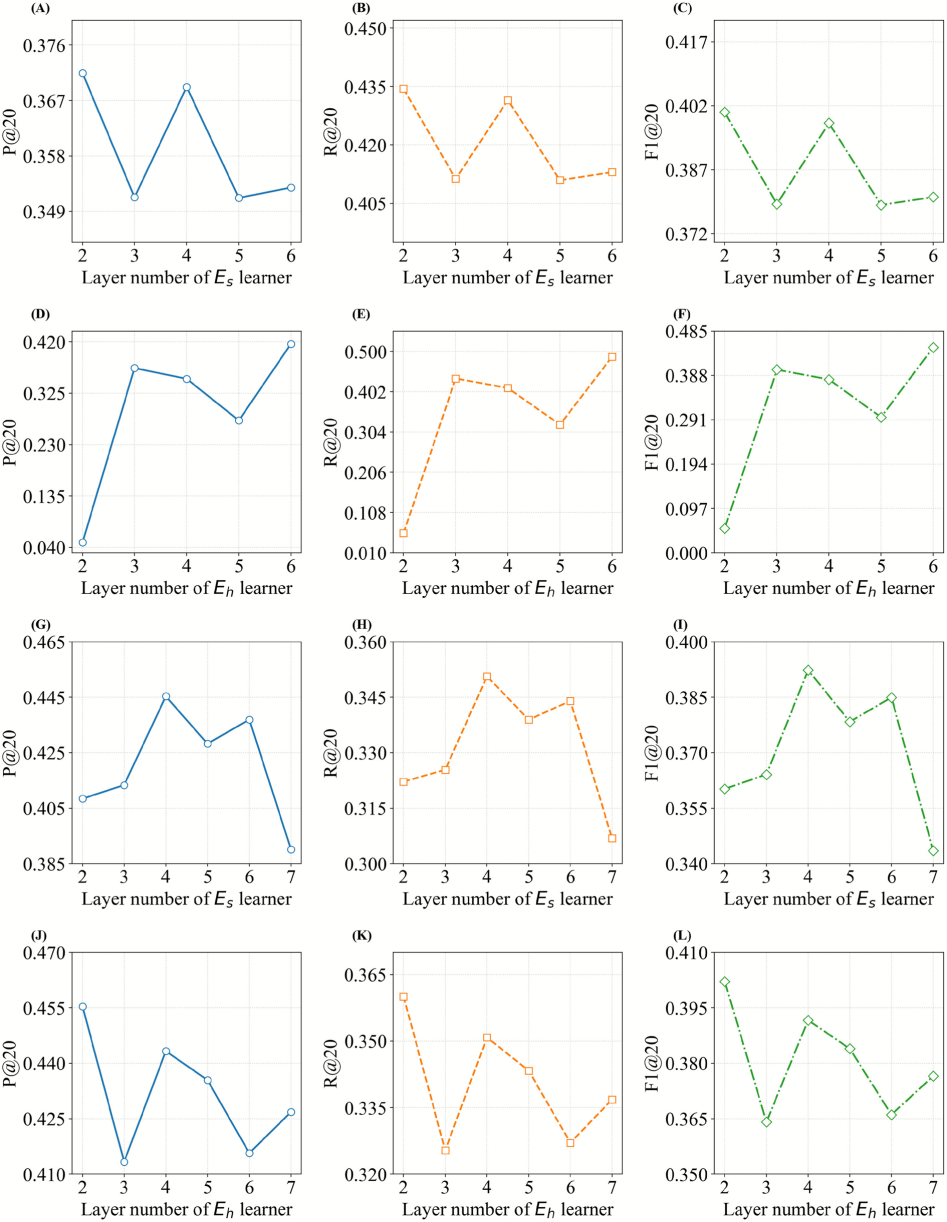
Performance of layer number of MLP on *TCM-Lung* and *TCM-SSD*. (**A**-**F**) *TCM-Lung* dataset: (**A**-**C**) E_s_ MLP depth, (**D**-**F**) E_h_ MLP depth. (**G**-**L**) *TCM-SSD* dataset: (**G**-**I**) E_s_ MLP depth, (**J**-**L**)  E_h_ MLP depth.

To visually evaluate the effect of the number of MLP layers in HRM on model performance, we tuned the number of MLP layers, investigating their effect on the model’s performance. In optimizing the number of MLP layers to enhance the precision of HPR, we kept the $E_h$ MLP fixed while adjusting the $E_s$ MLP to study their impact on recommendation performance. The optimal results were achieved with 5 layers for $E_h$ MLP and 7 layers for $E_s$ MLP, yielding P@5 of 60.25%, R@5 of 17.75%, and F1@5 of 27.42%. Fig 6A–C shows that with 3 $E_h$ MLP layers, increasing the $E_s$ MLP layers causes the performance of PresRecCDL to fluctuate and decrease. In Fig. 6D–F, with 2 layers for $E_s$ MLP, increasing the performance of $E_h$ MLP. These results indicate that PresRecCDL performs better when the number of $E_s$ MLP layers is greater than $E_h$ MLP layers. In Fig. 6G–L, as the MLP layers for both embeddings increase, the performance decreases in a fluctuating manner.

### Network pharmacology based case study

To show the effectiveness of PresRecCDL, we conduct a case study. We predicted that the right herbs are irkutsk Anemone Rhizome, tangshen, roasted rhizoma atractylodis, heartleaf houttuynia herb, lesser galangal rhizome, and danshen root. Table 2 is shown, where Symptom set is the set of symptoms of the patient, PresRecCDL is the herb predicted by the model, and ground truth is the true herb. The results demonstrate that the results predicted by PresRecCDL can cover more ground truth and reach 0.60 on P@10, 0.50 on R@10, and 0.55 on F1@10. It can be inferred from the above cases that our method exhibits a good performance. From the results, the proposed model predicted 24 herbs correctly on *TCM-SSD* and 15 herbs correctly on *TCM-Lung*, while TCMPR only predicted 4 herbs and 1 herb correctly on the same dataset.

**Table 2:** Cases on PresRecCDL.

Index	Symptom set	Herbal prescription set		
		TCMPR	PresRecCDL	Ground truth
Case 1	眠差、心慌、胸闷、胃 痛、双肺呼吸音粗、咳 痰、气喘、反酸、乏力 、发热、胃胀	甘草、半夏、茯苓、陈 皮、白芍、白术、黄芩 、柴胡、黄芪、当归、党 参、枳壳、丹参、麦冬* 、赤芍、杏仁、桂枝、 太子参、枳实、姜	醋莪术通草*、桔梗*、墨 旱莲*、麦冬*、苦杏仁*、 龙葵*、白鲜皮*、款冬花* 、诃子*、蜜紫菀*、粉葛* 、淫羊藿、麦芽*、麸炒苍 术*、石斛、铁线透骨草* 、锁阳、当归、紫苏子*、 制吴茱萸	苦杏仁*、款冬花*、紫苏 子*、桔梗*、金荞麦*、麦 冬*、麦芽*、豆蔻*、白鲜 皮*、墨旱莲*、人工牛黄* 、麸炒苍术*、龙葵*、生 山楂*、粉葛*、炙乳香*、 铁线透骨草*、诃子*、醋 莪术通草*、蜜紫菀*、蜜 款冬花*
Case 2	眠差、心慌、胸闷、胃 痛、双肺呼吸音粗、咳 痰、气喘、反酸、乏力 、发热、胃胀、头晕、 汗出、体亏虚、呼吸困 难、咳嗽、纳差	甘草、黄芩*、桔梗、杏 仁、半夏*、柴胡、连翘* 、茯苓、陈皮、麻黄、 大黄、白芍、枳壳、麦 冬、石膏、赤芍、薄荷 、玄参*、防风、白术	款冬花*、清半夏*、连翘* 、香附*、龙葵*、苦杏仁* 、玄参*、半夏*、黄芩*、 川楝子*、山药*、浙贝母* 、化橘红*、龙骨*、南沙 参*、北柴胡*、荷叶*、绵 萆薢*、枇杷叶、巴戟天* 、牛蒡子*、甘松*、麸炒 苍术、厚朴*、蜜槐角、 菟丝子*、肉桂、白芍、 鱼腥草、煅磁石*	黄芩*、太子参*、苦杏仁* 、化橘红*、款冬花*、射 干*、牛蒡子*、川楝子*、 北柴胡*、鱼腥草*、桑白 皮*、山药*、玄参*、薏苡 仁*、南沙参*、半夏*、香 附*、巴戟天*、绵萆薢*、 菟丝子*、连翘*、厚朴*、 龙骨*、猪苓*、人参*、紫 河车*、蛤壳*、荷叶*、猫 爪草*、甘松*、浙贝母*、 清半夏*、龙葵*、蒲公英 通草*、煅磁石*

The symbol * indicates that the herb recommended by relevant model is consistent with those prescribed by the doctor

The English translations for the symptom names mentioned in the case are as follows: 眠差(Sleep deprivation),心慌(palpitation),胸闷(oppression in chest),胃痛(stomach pain),双肺呼吸音粗(coarse breath sounds bilaterally),咳痰(coughing of phlegm),气喘(asthma),反酸(acid regurgitation),乏力(fatigue),发热(fever),胃胀(stomach distension),头晕(dizziness),汗出(perspiration),体亏虚(dyspnea),呼吸困难(difficulty breathing),咳嗽(cough),纳差(poor appetite)

The English translations for the Chinese herbal names mentioned in the case are as follows: 诃子(medicine terminalia fruit),锁阳(songaria cynomorium herb),豆蔻(Cardamom),麦冬(germinated barley),荷叶(lotus leaf),鱼腥草(heartleaf houttuynia herb),半夏(pinellia tuber),紫河车(human placenta),铁线透骨草(Achyranthis Bidentatae Radix),川楝子(szechwan chinaberry fruit),石膏(gypsum),太子参(heterophylly falsestarwort root),龙葵(Dragon Arum),白鲜皮(densefruit pittany root-bark),粉葛(Kudzu),山药(common yam rhizome),桂枝(cassia twig),蜜紫菀(Sweet Violet),清半夏(alum processed pinellia [tuber]),人参(ginseng),白术(largehead atractylodes rhizome),炙乳香(Frankincense Resin),巴戟天(morinda root),猪苓(zhuling),苦杏仁(bitter apricot seed),桑白皮(Cortex Mori),桔梗(platycodon root),醋莪术通草(Vinegar Atractylodes and Glabrous Greenbrier Rhizome),甘松(nardostachys root),金荞麦(golden buckwheat rhizome),款冬花(common coltsfoot flower),杏仁(bitter apricot seed),丹参(danshen root),姜(ginger),蒲公英通草(Dandelion Herb),牛蒡子(great burdock achene),大黄(rhubarb root and rhizome),白芍(debark peony root),麸炒苍术(Cang Zhu (Atractylodis Rhizoma)),茯苓(Indian bread),当归(Chinese angelica),煅磁石(Calcined Magnetite),薄荷(mint),猫爪草(catclaw buttercup root),防风(divaricate saposhnikovia root),射干(blackberry lily rhizome),蛤壳(clam shell),人工牛黄(Synthetic Musk),薏苡仁(coix seed),石斛(dendrobium),生山楂(Fresh Hawthorn),南沙参(fourleaf ladybell root),枇杷叶(loquat leaf),蜜槐角(Honey Locust Thorn),黄芩(baical skullcap root),枳实(immature orange fruit),厚朴(officinal magnolia bark),柴胡(Chinese thorowax root),北柴胡(Radix Bupleuri),制吴茱萸(Processed Evodia Fruit),墨旱莲(yerbadetajo herb),甘草(liquorice root),枳壳(orange fruit),肉桂(cassia bark),绵萆薢(Chinese Dodder Seed),紫苏子(perilla fruit),蜜款冬花(Honeyed Osmanthus),淫羊藿(epimedium herb),浙贝母(thunberbg fritillary bulb),菟丝子(dodder seed),黄芪(milkvetch root),龙骨(bone fossil of big mammals),香附(nutgrass galingale rhizome),赤芍(peony root),党参(tangshen),化橘红(pummelo peel),连翘(weeping forsythia capsule),麻黄(ephedra), 麦芽(germinated barley),玄参(figwort root),陈皮(dried tangerine peel)

To further demonstrate the effectiveness of our model results, we performed network analyses for the two cases above using network pharmacology techniques. The network data we used, including the relationships between symptoms and genes and the relationships between herb and targets, were derived from the SymMap [46] database. Additionally, we mapped the genes related to symptoms and herbs onto the STRING-11.0 [51] protein–protein interaction network to observe the associations between symptom-related proteins and herb-related target proteins.

**Fig. 7:**
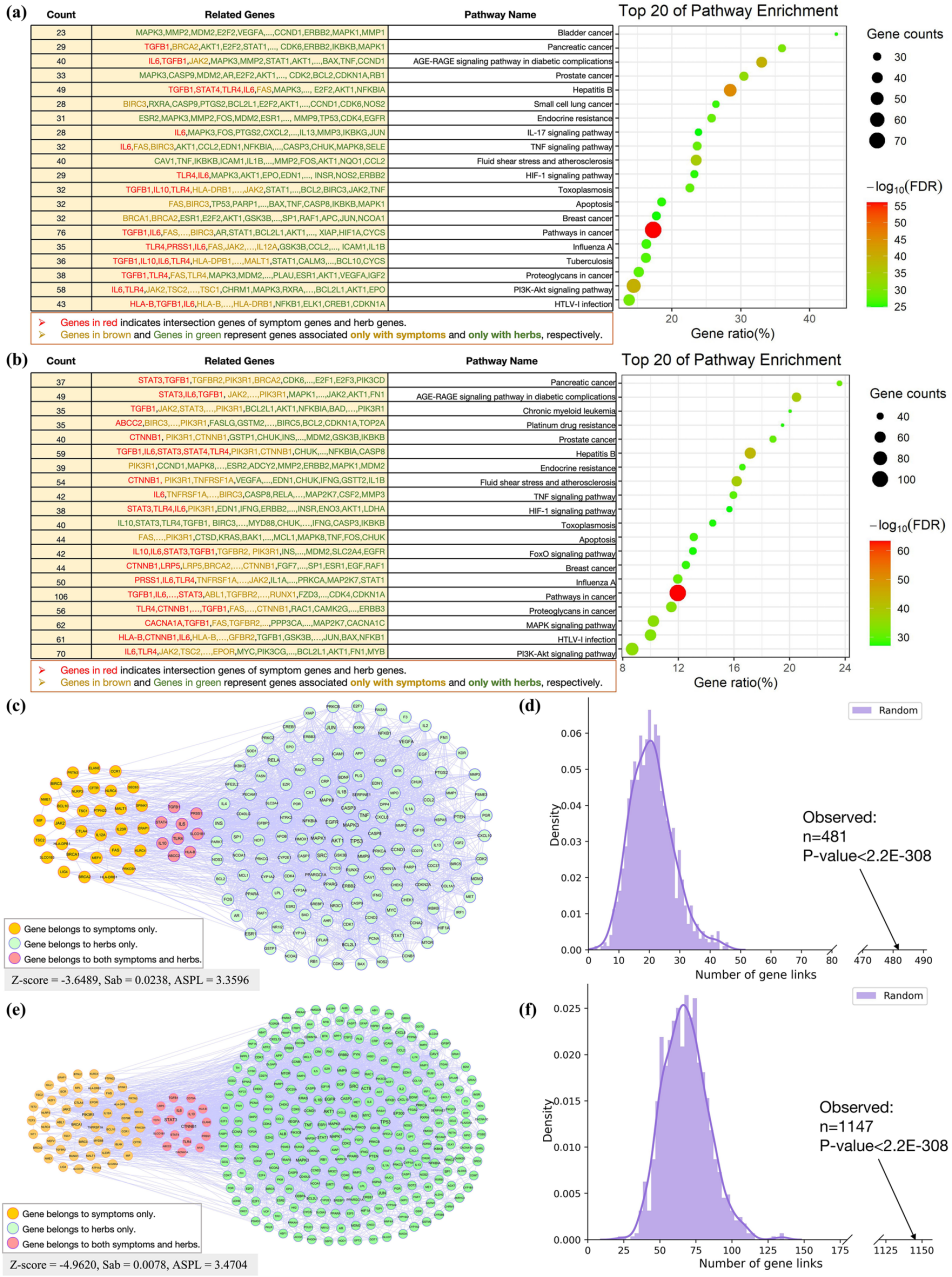
Case study on PresRecCDL with the view of network pharmacology. **a** Pathway enrichment analysis of the intersection genes for both symptom and herb genes associated with Case 1. The bubble chart on the right illustrates significantly enriched pathways related to Case 1. The list on the left details the enriched genes, including those from the symptom genes and predicted genes of herbs. **b** Pathway enrichment analysis of the intersection genes for both symptom and herb genes associated with Case 2. **c** Visualization of links of symptom genes and herb targets for Case 1 on the PPI network. **d** The observed number of network links is significantly larger than the random control (*P* = 0.0, binomial test) for Case 1. **e** Visualization of links of symptom genes and herb targets for Case 2 on the PPI network. **f** The observed number of network links is significantly larger than the random control (*P* = 0.0, binomial test) for Case 2.

Fig. 7a, b show pathway enrichment analysis of the intersection genes for both symptom and herb genes associated with Case 1 and Case 2, respectively. The bubble chart on the right illustrates significantly enriched pathways. The list on the left details the enriched genes, including those from the symptom genes and predicted genes of herbs. The top 20 pathways in both Case 1 and Case 2 include *pathways in cancer* [52], *Hepatitis B* [53], and *AGE-RAGE signaling pathway in diabetic complications*[54], among others. These pathway results suggest that the predicted herbs are closely related to the diseases reflected by the symptoms.

Fig. 7c illustrates the protein–protein interaction network formed by the genes corresponding to the symptoms and herbs in Case 1. The figure highlights the Z-score, $S_{AB}$, and ASPL results. The analysis shows a strong association between the proteins corresponding to the model-recommended herbs and the genes corresponding to the symptoms. Specifically, Z-score = −3.6489, $S_{AB}$ = 0.0238, and ASPL = 3.3596. These findings indicate a significant molecular-level association between the predicted herbs and the target symptoms. Furthermore, the observed number of network links is significantly larger than the random control (P = 0.0, binomial test) for Case 1, as shown in Fig. 7d.

Fig. 7e illustrates the protein–protein interaction network for Case 2, showing the association between the genes corresponding to the symptoms and herbs. The Z-score, $S_{AB}$, and ASPL values for this case are − 4.9420, 0.0078, and 3.4704, respectively. The association is higher than in Case 1, and the genes corresponding to the herbs still exhibit a certain degree of network relevance with the symptom-related target proteins. Furthermore, the analysis of gene functional enrichment reveals the top 10 pathways (as shown in Fig. 7b), further supporting the scientific validity of the relevance of the herbs predicted by the model for symptoms. Furthermore, the observed number of network links is significantly larger than the random control (*P* = 0.0, binomial test) for Case 2, as shown in Fig. 7f.

## Discussion

In this section, we discussed the strengths and weaknesses of the proposed PresRecCDL and our future work.

### Advantages and prospects

In this study, we introduced PresRecCDL, a novel framework for herbal prescription recommendation. This framework comprises two key components: cross-domain learning and neural collaborative filtering. The cross-domain learning is adept at learning noise-reduced cross-domain features of herbs and symptoms in a unified space. Meanwhile, the neural collaborative filtering is tailored for a prescription recommendation, to enhance the precision and efficacy of recommendations through the adept integration of cross-domain features. The proposed approach marks a departure from conventional recommendation systems, which are often constrained within single domains. By transcending these limitations, our framework opens up new avenues for HPR, offering fresh insights and methodologies to the field.

The introduction of PresRecCDL addresses several key challenges in TCM systems. First, it effectively mitigates the issue of data sparsity, which is prevalent in TCM datasets. By leveraging cross-domain learning, PresRecCDL enhances the robustness of recommendations, as demonstrated by improved precision (P@5 at 60.26%) and recall (R@5 at 17.71%). Second, the integration of cross-domain features has proven valuable, with experimental results confirming that a combination of $E_s$ learner and $E_h$ learner yields significant performance improvements. This not only enhances the expected outcomes but also provides a more nuanced understanding of symptom-herb relationships.

Moreover, PresRecCDL demonstrates that cross-domain learning and collaborative filtering techniques can be effectively integrated to advance both research and clinical applications within TCM. The model’s ability to utilize comprehensive embeddings of symptoms and herbs supports more informed decision-making, potentially improving patient outcomes. The case study based on network pharmacology provides strong evidence for the effectiveness of our approach, particularly its rationality and reliability at the molecular mechanism level. In summary, our study underscores the advantages of PresRecCDL in addressing data sparsity, validating the value of cross-domain features, and integrating advanced learning techniques in the field of traditional medicine.

### Limitations and future work

On one hand, although the proposed model effectively integrates data from different disease categories for cross-domain learning, thereby enhancing its performance across various disease types, it does not consider the dosage and compatibility information of herbal medicines. Dosage and the compatibility of herbal medicines are crucial for the treatment of patient symptoms using TCM prescriptions, posing a significant challenge in existing research. In future studies, we aim to incorporate dosage and compatibility information into our model design.

On the other hand, the proposed model does not incorporate TCM elements such as pattern identification (syndrome differentiation), treatment principles, and treatment methods, which are essential for constructing intelligent clinical decision support systems in TCM. However, the field currently lacks high-quality publicly available clinical datasets, particularly those containing these elements. In future work, we plan to address this gap by constructing datasets that include these elements and leveraging them in the design of our model. By doing so, we aim to achieve more effective prescription recommendations.

## Conclusion

We introduced a novel framework for herbal prescription recommendation termed PresRecCDL. This framework comprises cross-domain neural collaborative filtering, aiming to enhance recommendation accuracy and effectiveness by effectively integrating cross-domain features. Our research breaks through the limitations of previous HPR models confined to single domains. In the future, we will focus on constructing HPR models for real-world TCM applications. Specially, we will consider herb dosage information to achieve a more realistic and contextually informed intelligent herbal prescription recommendation.

## Data Availability

The source codes and the data of PresRecCDL are available at https://github.com/2020MEAI/PresRecCDL.

## Abbreviations

ASPL: Average shortest path length
CBCDR: Content-based cross-domain recommendation
CDR: Cross-domain recommendation
CFCDR: Collaborative filtering-based cross-domain recommendation
CPM: Correlation pattern predictor module
HCM: Herb cross-domain module
HPR: Herbal prescription recommendation
HRM: Herbal recommendation module
MF: Matrix factorization
MLP: Multi-layer perceptron
NR: Network Relevance
OR: Odd ratio
SCM: Symptom cross-domain module
TCM: Traditional chinese medicine
TCM-Lung: the Lung disease dataset
TCM-SSD: the stomach and spleen disease dataset

## Footnotes

1 Sample-level sparsity describes the proportion of unobserved symptoms or herbs at the sample level, and can be formulated as: $\text {Sparsity}_{sample-s}=1 - \frac{1}{N}\sum _{i=1}^{N}\frac{|S_i|}{|S|} \quad \text {, Sparsity}_{sample-h}=\quad 1 - \frac{1}{N}\sum _{i=1}^{N}\frac{|H_i|}{|H|}.$ where the dataset contain *N* samples, each sample *i* includes a subset of symptoms $S_i \subseteq S$ and a subset of herbs $H_i \subseteq H$.
